# Evaluation of capillary leakage after vasopressin resuscitation in a hemorrhagic shock model

**DOI:** 10.1186/s13017-018-0172-7

**Published:** 2018-03-05

**Authors:** Roberto Bini, Osvaldo Chiara, Stefania Cimbanassi, Giorgio Olivero, Antonella Trombetta, Paolo Cotogni

**Affiliations:** 1 Department of Surgery, S. Giovanni Bosco Hospital, Turin, Italy; 2 Trauma Center and Metropolitan Trauma Network Department, Niguarda Hospital, Milan, Italy; 3 Department of Surgical Sciences, S. Giovanni Battista Hospital, University of Turin, Turin, Italy; 4 Department of Medical Sciences, University of Turin, Turin, Italy; 5 Department of Anesthesia and Intensive Care, S. Giovanni Battista Hospital, University of Turin, Via Giovanni Giolitti 9, 10123 Turin, Italy

**Keywords:** Hemorrhage, Shock treatment, Arginine vasopressin, Vasopressors

## Abstract

**Background:**

Hemorrhagic shock (HS) is a major threat to patients with trauma and spontaneous bleeding. The aim of the study was to investigate early effects of vasopressin on metabolic and hemodynamic parameters and endothelium permeability by measuring capillary leakage compared to those of other resuscitation strategies in a HS model.

**Methods:**

Forty-five Sprague-Dawley rats were randomized into five groups: S group (*n* = 5), sham-operated rats without shock or resuscitation; HS group (*n* = 10), HS and no resuscitation; RL group (*n* = 10), HS and resuscitation with Ringer’s lactate (RL); RLB group (*n* = 10), HS and resuscitation with two-third shed blood plus RL; and vasopressin group (*n* = 10), HS and resuscitation with RL, followed by continuous infusion of 0.04 U/kg/min vasopressin. The effects of resuscitation on hemodynamic parameters [mean arterial pressure (MAP), superior mesenteric artery blood flow (MBF), and mesenteric vascular resistances (MVR)], arterial blood gases, bicarbonate, base deficit, and lactate levels as well as on capillary leakage in the lung, ileum, and kidney were investigated. Capillary leakage was evaluated with Evans blue dye extravasation.

**Results:**

In the vasopressin group, the MAP was higher than in the RL and RLB groups (*p* < 0.001), while MBF was decreased (*p* < 0.001). MVR were increased only in the vasopressin group (*p* < 0.001). Capillary leakage was increased in the lungs of the animals in the vasopressin group compared to that in the lungs of animals in the RLB group (*p* < 0.05); this increase was associated with the lowest partial pressure of oxygen (*p* < 0.05). Conversely, decreased capillary leakage was observed with vasopressin in the ileum (*p* < 0.05). Increased capillary leakage was observed in the kidney in the RLB and vasopressin groups (*p* < 0.05). Lastly, vasopressin use was associated with higher base deficit and lactate levels when compared to the RL and RLB groups (*p* < 0.001).

**Conclusion:**

Although vasopressin was proposed as a vasoactive drug for provisional hemodynamic optimization in the early phase of HS resuscitation, the overall findings of this experimental study focus on the possible critical side effects of vasopressin on metabolic parameters and endothelium permeability.

## Background

Hemorrhagic shock (HS) represents a major threat to trauma and spontaneous bleeding patients [1]. Organ dysfunction and/or failure may be consequences of HS and account for high mortality, despite adequate fluid resuscitation and subsequent restoration of normotension. There is an ongoing discussion on strategies used for resuscitation of trauma patients with HS [2]. Bleeding control and maintenance of tissue oxygenation with fluid resuscitation remain the mainstays of therapy for patients with HS. However, severe and protracted hypotension with decreased tissue perfusion induced by an ongoing hemorrhage may progress in a condition of profound shock, making the patient unresponsive to fluid resuscitation and catecholamines. Correction of hypovolemia does not resolve hypotension in these patients because of the occurrence of peripheral vasodilatation. Landry and Oliver [3] suggested the following three mechanisms that have been implicated in the pathogenesis of vasodilatory shock, which occurs during the late phase of HS: (a) activation of ATP-sensitive potassium channels in the plasma membrane of the vascular smooth muscles, (b) activation of the inducible form of nitric oxide synthase, and (c) deficiency of the hormone vasopressin. The last mechanism led to the investigation of the role of vasopressin in the treatment of advanced stages of HS [4].

Recent animal studies have shown that vasopressin treatment achieves hemodynamic optimization during the early phase of HS (mimicking the prehospital phase) while fluids and catecholamines showed neither improvement of hemodynamic parameters nor survival [5, 6]. However, vasopressin use has been associated with some adverse effects such as ischemic complications particularly in the splanchnic and skin circulation [7]. Moreover, a multicenter prospective cohort study suggested that the early use of vasopressors for hemodynamic improvement after HS when compared to aggressive volume resuscitation was deleterious and should be cautiously used, supporting the concept of “caution before constriction” [8]. Collier et al. found that vasopressin use was associated with increased mortality in trauma patients with shock [9].

It has been demonstrated that intense inflammatory response may develop after HS [10]. Massive blood loss and resuscitation cause a global ischemia-reperfusion effect with an upregulation of cytokine expression [11] and oxidative and nitrosative stresses [12] that may lead to organ damage and dysfunction. During advanced stages of HS, changes in capillary permeability with leak and plasma extravasation (i.e., capillary leakage) into the interstitial space contribute to microcirculatory disturbances and organ damage [13] with edema formation [14].

The aim of this study was to investigate the early effects of vasopressin on metabolic and hemodynamic parameters and endothelium permeability by measuring capillary leakage compared to those of other resuscitation strategies in a HS model.

## Methods

### Animal preparation and surgical procedure

All experiments were conducted in accordance with the requirements indicated in the guidelines of Institutional Animal Care and Use Committees (IACUC), after the approval from the Ethics Committee of the Piedmont Regulatory Agency for Care and Use of Laboratory Animals. Male Sprague-Dawley rats (weight range, 290–350 g; median weight, 325 g; age range, 50–65 days) were used (Charles River, Calco, Como, Italy). The animals were housed individually and allowed free access to food and water. The animal rooms were windowless with controlled temperature (22 ± 2 °C) and lighting (12-h light/dark cycle), where the rats were allowed to acclimatize for a minimum of 5 days after arrival at the laboratory. Subsequently, the animals were anesthetized with ketamine plus xylazine intramuscular (90 and 10 mg/kg body weight [b.w.], respectively) and restrained in a supine position. Body temperature was monitored by a rectal thermal probe and maintained at 37 °C with a heating pad. The left groin was shaved and prepped with povidone-iodine solution. The femoral artery was dissected using a minimal dissection technique, distally ligated and cannulated with a 22-gauge heparinized catheter. A blood pressure transducer (Isotec-Healthdyne Cardiovascular, Marietta, GA) and a monitor allowed for the continuous monitoring of mean arterial pressure (MAP). A midline laparotomy was performed, and superior mesenteric artery blood flow (MBF) was measured with a perivascular probe (T206, Transonic Systems, Ithaca, NY). Blood samples were collected from the arterial catheter. Mesenteric vascular resistances (MVR) were calculated as MAP/MBF. Arterial blood gases, bicarbonate, base deficit, and lactate levels were measured with a blood-gas analyzer (ABL System 500, Radiometer Medical, Copenhagen, Denmark).

### Experimental protocol

**Fig. 1:**
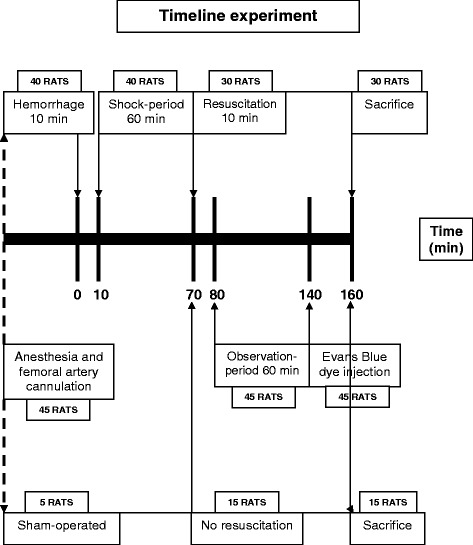
Timeline experiment. Forty-five rats underwent anesthesia and artery cannulation. Forty rats were subjected to pressure-controlled hemorrhage over a 10-min period, while five rats were sham-operated. The target value of hemorrhagic shock was a reduction in mean arterial pressure (MAP) to 35–40 mmHg. The constant MAP was maintained for 60 min (shock period). After 1 h of shock, 30 hemorrhaged rats were resuscitated over a 10-min period according to the experimental protocol. One hour after the end of resuscitation (observation period), Evans blue dye was injected. Twenty minutes after Evans blue injection, all the rats were euthanized

Forty-five rats were randomized into the following five groups: S group (*n* = 5), sham-operated (S) without shock or resuscitation (anesthesia and artery cannulation only); HS group (*n* = 10), HS and no resuscitation; RL group (*n* = 10), HS and resuscitation with Ringer’s lactate (RL) intravenously; RLB group (*n* = 10), HS and resuscitation with two-third shed blood (i.e., 2/3 the hemorrhage volume) plus RL; and vasopressin group (*n* = 10), HS and resuscitation with RL, followed by continuous infusion of 0.04 U/kg/min vasopressin via a syringe pump (5 ml/h). The experiment was carried out using a model with pressure-controlled hemorrhage previously described [15] (Fig. 1). Briefly, the experiment began by gradually bleeding each rat, except those in the S group, by withdrawing blood from a catheter inserted into the femoral artery over a 10-min period. The target value of HS was a reduction in MAP to 35–40 mmHg. The constant MAP was maintained for 60 min with further withdrawal or infusion of small amounts of shed blood. The blood was collected in a heparinized tube and incubated at 37 °C. After 1 h of shock, hemorrhaged rats, except those in the HS group, were resuscitated with the same volume of fluids or shed blood (11.3 ± 0.7 ml) over a 10-min period according to the experimental protocol. Afterward, all the rats underwent the same rate of fluid infusion (5 ml/h).

Monitoring after anesthesia recovery included attention to specific criteria for evaluating the signs of postoperative pain and distress. The rats were assessed clinically to judge the efficacy of the analgesia, and an analgesic was administered in the presence of behavioral signs of pain or distress (rapid breathing, abnormal movement or posture/positioning, twitching/trembling) according to our institutionally approved animal protocol developed through veterinary consultation. Postoperative analgesia (ketamine, 80 mg/kg b.w., intramuscular) was maintained throughout the experimental period until the animals were euthanized. In relation to the type and duration of surgical procedure, no animals showed any clinically apparent signs of pain or distress with a single postoperative dose of ketamine.

Effects of resuscitation on capillary leakage in the lung, ileum, and kidney were investigated in the three treatment groups and compared to the HS group. Capillary leakage was evaluated with Evans blue (EB) dye extravasation using the following method: 1 h after the end of resuscitation, 50 mg/kg b.w. of EB dye was injected as previously described by Schumacher et al. [16]. Twenty minutes after EB injection, the rats were euthanized with an overdose of sodium pentobarbital (intravenously; 150 mg/kg b.w.). The inferior vena cava was incised to exsanguinate the animals. An 18-gauge catheter was placed into the apex of the left ventricle with its tip inside the aorta in order to perfuse the circulatory system with 0.9% NaCl containing heparin (100 U/ml). The perfusion volume (at a perfusion pressure of 40 mmHg) was four times the calculated blood volume of rat (7.46 ml/100 g b.w.). This procedure allows the removal of excess intravascular dye following which the lungs, kidneys, and ileum were dissected and removed. The tissues were rinsed in saline, gently blotted, and weighed. Half of each tissue was dried by incubation at 60 °C for 48 h. EB was extracted from the remaining halves by incubation in formamide (4 ml/g tissue; 99.5%, F-7503, Sigma Aldrich Chemie, Germany) at room temperature for 48 h. The supernatant was removed from the tissues and read against a formamide blank at 620 nm (Spectrophotometer, Beckmann DU 7400, Germany). Absorbance was compared to a standard curve of 0.05–50 μg ml^−1^ EB in formamide. Extravasation was expressed as microgram EB per gram dry tissue. The blood samples and parameters (MAP, MBF, and MVR) were obtained at 0′ (at the start of the experiment), 10′ (end of shock period), and 140′ (at 1 h after the end of resuscitation). The collection of blood samples (hemoglobin, partial pressure of oxygen, bicarbonates, base deficit, and lactate) and tissue specimens (lung, ileum, and kidney) in the S and HS groups was time-matched to the resuscitation groups.

### Statistical analysis

The hemodynamic and metabolic variables were expressed as mean ± standard deviation. Multiple comparisons between groups of parametric data were carried out using one-way ANOVA, followed by the Bonferroni post hoc test. The level of significance was defined as a *p* value < 0.05. Data were analyzed by R 3.3.2 (R Foundation for Statistical Computing, Vienna, Austria, http://www.r-project.org).

## Results

### Hemodynamic parameters

**Fig. 2:**
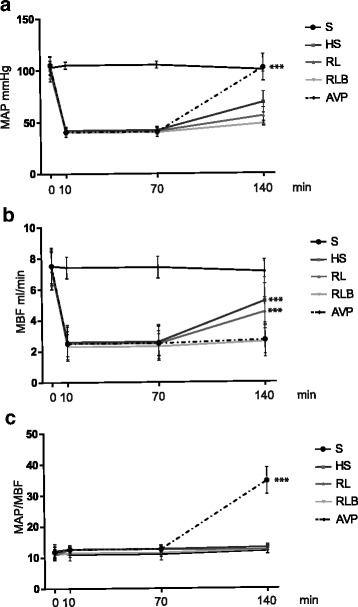
Hemodynamic parameters. Data are represented as mean ± SD of mean arterial pressure (MAP) expressed as mmHg (**a**), mesenteric arterial blood flow (MBF) expressed as milliliters per minute (**b**), and MAP/MBF ratio (mmHg ml^−1^ min) (**c**). S, sham-operated; HS, hemorrhagic shock; RL, resuscitation with Ringer’s lactate; RLB, resuscitation with Ringer’s lactate and blood; AVP, resuscitation with Ringer’s lactate and vasopressin infusion. **a** ***p < 0.001 AVP vs RL and RLB; **b** ***p < 0.001 RL and RLB vs AVP; **c** ***p < 0.001 AVP vs all

The amount of shed blood was 8.0 ± 0.7 ml (26 ± 3.1 ml/kg b.w.); no differences in total blood loss were observed between the groups. MAP decreased from 105 ± 10 to 40 ± 4 mmHg after blood withdrawal (10′) in the hemorrhaged rats. At 140′, the MAP was higher in the vasopressin group than in the RL and RLB groups (102 ± 12.9, 55.5 ± 8.0, and 68.5 ± 10.3 mmHg, respectively, *p* < 0.001) (Fig. 2a).

At 10′, there were no differences in MBF among the hemorrhaged groups. At 140′, the MBF was higher in the RL and RLB groups when compared to the vasopressin group (4.5 ± 0.5, 5.2 ± 0.6, and 2.7 ± 0.3 ml/min, respectively, *p* < 0.001) (Fig. 2b).

At 10′, no differences in MVR (i.e., MAB/MBF) among the hemorrhaged groups were noted. At 140′, the MVR were increased only in the vasopressin group (34.5 ± 4.2 mmHg ml^−1^ min, *p* < 0.001) and remained unmodified in the RLB (13.1 ± 1.0 mmHg ml^−1^ min) and RL groups (12.4 ± 1.4 mmHg ml^−1^ min) (Fig. 2c). No mortality occurred during the experimental period.

### Laboratory data

**Fig. 3:**
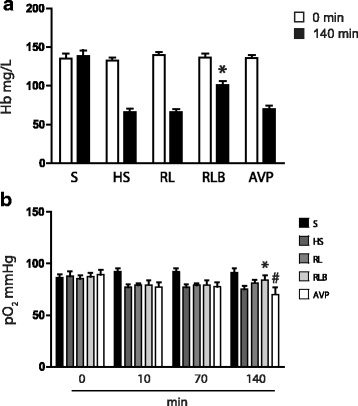
Hemoglobin and partial pressure of oxygen. Data are represented as mean ± SD of hemoglobin (Hb) concentration expressed as milligrams per liter (**a**) and partial pressure of oxygen (pO_2_) expressed as mmHg (**b**). S, sham-operated; HS, hemorrhagic shock; RL, resuscitation with Ringer’s lactate; RLB, resuscitation with Ringer’s lactate and blood; AVP, resuscitation with Ringer’s lactate and vasopressin infusion. **a** *p < 0.05 RLB vs HS, RL, and AVP; **b** *p < 0.05 RLB vs HS and RL, ^#^*p* < 0.05 AVP vs all

Hemoglobin was decreased after hemorrhage and partially recovered at 140′ only in the RLB group (*p <* 0.05) (Fig. 3a). Likewise, the partial pressure of oxygen (pO_2_) was partially recovered at 140′ in the RLB group only (*p <* 0.05). The pO_2_ was significantly lowest in the vasopressin group when compared to the other groups (*p <* 0.05) (Fig. 3b).

**Fig. 4:**
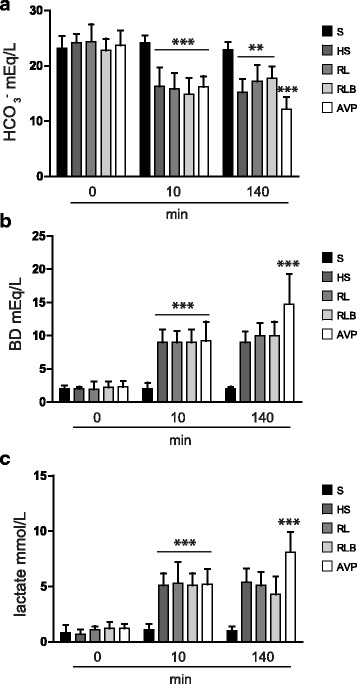
Bicarbonates, base deficit, and lactate. Data are represented as mean ± SD of bicarbonate (HCO_3_^−^) levels expressed as milliequivalents per liter (**a**), base deficit (BD) levels expressed as mEq/l (**b**), and lactate levels expressed as millimoles per liter (**c**). S, sham-operated; HS, hemorrhagic shock; RL, resuscitation with Ringer’s lactate; RLB, resuscitation with Ringer’s lactate and blood; AVP, resuscitation with Ringer’s lactate and vasopressin infusion. **a** ***p < 0.001 S vs all at 10′, **p < 0.01 HS, RL, and RLB vs S at 140′, ***p < 0.001 AVP vs all at 140′; **b** ***p < 0.001 S vs all at 10′, ***p < 0.001 AVP vs all at 140′; **c** ***p < 0.001 S vs all at 10′, ***p < 0.001 AVP vs all at 140′

At 10′, bicarbonate levels were decreased in all the groups (Fig. 4a), while base deficit (Fig. 4b) and lactate levels (Fig. 4c) were increased (*p* < 0.001). At 140′, bicarbonate levels were persistently decreased in the HS, RL, and RLB groups (*p* < 0.01), and the vasopressin group (*p* < 0.001). A significant increase in base deficit was observed in the vasopressin group at 140′ when compared to the RL and RLB groups (*p* < 0.001). Furthermore, lactate levels were higher in the vasopressin group when compared to the RL and RLB groups at 140′ (*p* < 0.001).

### Capillary leakage

**Fig. 5:**
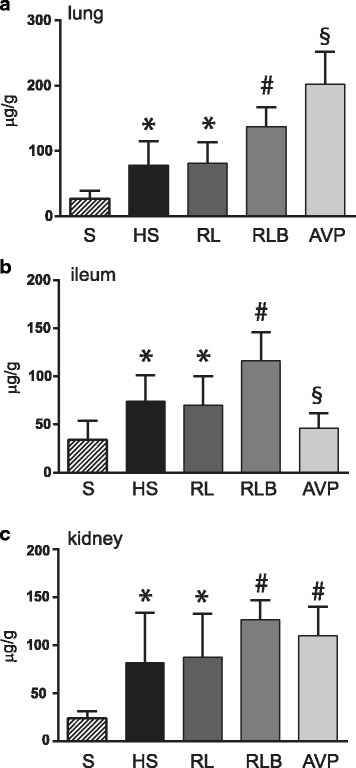
Capillary leakage in the lung (**a**), ileum (**b**), and kidney (**c**). Data are expressed as mean ± SD of micrograms of Evans blue (EB) per gram of dried tissue. HS, hemorrhagic shock; RL, resuscitation with Ringer’s lactate; RLB, resuscitation with Ringer’s lactate and blood; AVP, resuscitation with Ringer’s lactate and vasopressin infusion. **a** *p < 0.05 HS and RL vs S; ^#^*p* < 0.05 RLB vs HS and RL; ^§^*p* < 0.01 AVP vs HS and RL and *p* < 0.05 AVP vs RLB; **b** *p < 0.05 HS and RL vs S; ^#^*p* < 0.05 RLB vs HS and RL; ^§^*p* < 0.05 AVP vs HS and RL and *p* < 0.01 AVP vs RLB; **c** *p < 0.05 HS and RL vs S; ^#^*p* < 0.05 RLB and AVP vs HS and RL

Capillary leakage, evaluated as EB extravasation, was increased in the lung, ileum, and kidney in the HS group when compared to the S group (*p* < 0.05). In the lung, increased capillary leakage was observed in the RLB group when compared to the HS and RL groups (*p* < 0.05) and in the vasopressin group when compared to the HS and RL (*p* < 0.01) and RLB (*p* < 0.05) groups (Fig. 5a). In the ileum, increased capillary leakage was noted in the RLB group (*p* < 0.05) when compared to the HS and RL groups, whereas in the vasopressin group, the leakage was decreased when compared to the HS and RL (*p* < 0.05) and RLB (*p* < 0.01) groups (Fig. 5b). Increased capillary leakage was observed in the kidney in the RLB and vasopressin groups when compared to the HS and RL groups (*p* < 0.05) (Fig. 5c).

## Discussion

Bleeding control, maintenance of tissue oxygenation with fluid resuscitation, coagulation support, and maintenance of normothermia remain the mainstays of therapy for patients with HS [2]. Maintenance of adequate perfusion pressure assists tissue oxygenation and reduces the risk of short- and long-term organ failure and sepsis. This practice has been challenged in both clinical trials and experimental models [17, 18]. After trauma, fluid infusion of large-volume normal saline or small-volume hypertonic saline solutions may result in side effects, such as tissue edema and re-bleeding with hemodynamic deterioration and increased mortality [19, 20].

The use of a vasopressor in case of vasodilatation or insufficient vasoconstriction is effective in restoring the hemodynamic parameters with adequate vital organ infusion, thereby reducing the need for continuous fluid infusion [21, 22]. Indeed, previous studies demonstrated increased survival when a small-volume fluid hypotensive resuscitation concept was used for penetrating trauma [23]. Several vasopressors including vasopressin have been studied in HS. Vasopressin acts via a number of receptors that are different from those of catecholamines. It exerts a direct vasoconstrictor effect on the systemic vascular smooth muscle by means of the vasopressin V1 receptors.

In our study, MAP was restored to baseline values only in the vasopressin group; administration of RL solution with or without blood did not obtain this effect. In the vasopressin group, we observed consistent vasoconstriction of the sub-diaphragmatic vascular bed, as demonstrated by the absence of improvement in MBF, leading to a significant increase in MVR. These results are consistent with those reported by Stadlbauer et al. [24].

In this study, we used an infusion rate of 0.04 U/kg/min which is much lower than the dose of vasopressin commonly used (i.e., 0.04 U/min) considering the rat median weight (i.e., 325 g). High amounts of vasopressin infusion were reported to be associated with detrimental side effects [8, 25].

Capillary permeability increases when there is damage or death of the endothelial cells and change in the intercellular space, rendering the endothelial cells and basement membrane less effective barriers to large molecules. Damage to the endothelial cells is initiated by an ischemia-reperfusion phenomenon, which promotes neutrophil activation and the release of proteases, oxygen radicals, and other toxic substances [26–28]. Various organs reveal different sensitivities to hypoxic-ischemic insult resulting in increased or decreased diffusion of macromolecules from the circulation into the interstitial space [29]. The initial phase of HS resuscitation comprises infusions of fluids, often leading to rapid tissue swelling, that may result in organ damage and dysfunction due to fluid and protein extravasation [30].

In the current study, HS alone was able to determine significant capillary leakage in the lung, ileum, and kidney, which was established by the albumin-bound EB extravasation in these organ tissues. Schumacher et al. also demonstrated significant capillary leakage in the lung and kidney in a similar HS experimental model [16]. In the vasopressin group, an increased capillary leakage, markedly higher than that in the group resuscitated with RL plus blood, was observed in the lung. This finding was also associated with a significant decrease in pO_2_ in this group.

According to the equation by Starling, increase in vasopressin-induced capillary hydrostatic pressure determines the increase in capillary leakage, in the presence of a drop in plasma oncotic pressure owing to dilution of circulatory volume, protein loss, and extravascular leakage [30]. This is consistent with the results of the study by Stadlbauer et al., which asserts that one of the effects of vasopressin may be the shifting of blood away from below the diaphragm to above it with increased extravascular lung water [24].

Contrarily, Lee et al. [31] showed that the vasopressin group had lower pulmonary edema than the group resuscitated with RL and blood; however, the different experimental protocol (pulmonary edema formation was investigated 3 days after HS) used may account for this inconsistency. Feinstein et al., in an animal model of thoracic trauma and HS, showed that the early use of vasopressin improves lung function when compared to animals treated with fluids only [32]. This finding is not in conflict with our data because in that study, the animals were mechanically ventilated, whereas the rats in our study maintained spontaneous breathing throughout the experimental procedure. Actually, the positive pressure may have exerted a reduction in alveolar edema.

Interestingly, resuscitation with RLB was associated with increased capillary leakage in the ileum, whereas vasopressin use was accompanied by a decrease in leakage. However, the consistent vasoconstriction of mesenteric blood flow induced by vasopressin observed in our study may impair the gut mucosal perfusion. Stadlbauer et al. showed that infusion of vasopressin was followed by transient non-bloody diarrhea 3 h after stabilization, suggesting a potential gut ischemia [33].

In the present study, a similar increase in capillary leakage was noted in the kidney in the RLB and vasopressin groups. In the extracerebral tissues, vasopressin works primarily on the arterioles with less constriction on the coronary and renal vessels, thereby having vasodilatory effects on cerebral and pulmonary flow [34]. Besides, preservation of renal blood flow by vasopressin has been demonstrated in an experimental model of HS [35, 36].

In experimental models, early administration of vasopressors was able to restore a quasi-normal hemodynamic status by mobilizing non-constraint venous blood volume and thus normalizing hemodynamic parameters (arterial blood pressure, cardiac output, and pulse pressure variations) [37]. This masked hypovolemia was associated with deleterious effects of vasopressor use in experimental hemorrhage models, due to the worsening of tissue hypoperfusion leading to metabolic disturbances, such as metabolic acidosis [38, 39].

Interestingly, in the present study, we found significantly higher base deficit and lactate levels in the vasopressin group when compared to the other groups. This finding recognizes a multifactorial etiology. Recently, in a swine model of HS, Soller et al. [40] found that lactate is a predictor of mortality with an area under the ROC curve of 0.87. Besides, several studies have already identified worsening base deficit as an indicator of increased transfusion requirement [41, 42]. Furthermore, base deficit has been associated with increased mortality and intensive care unit and in-hospital lengths of stay, with a higher incidence of shock-related complications such as acute respiratory distress syndrome, renal failure, hemocoagulative disorders, and multiorgan failure [43]. Monitoring of base deficit has also been suggested as an indicator and a monitoring parameter for the success of resuscitation efforts [44].

On a final note, our controlled hemorrhage and resuscitation model does a good job of mimicking clinical reality, but we should make a strong case for translation of this research to humans. According to a very recent review [45], in HS, a successful resuscitation requires to stanch all sources of hemorrhage and rapidly restore the intravascular volume by a resuscitation approach that emphasizes the use of all blood products. However, vasopressin supplementation is considered a therapy that may reduce blood product and fluid requirements in patients with HS [45, 46].

### Strengths and limitations of the study

To the best of our knowledge, this is the first study investigating the early effects of vasopressin on metabolic and hemodynamic parameters and endothelium permeability by measuring the capillary leakage in different organ tissues compared to those of other resuscitation strategies in a HS model.

This study has several limitations. First, we used a controlled hemorrhage model, not the same as free hemorrhage following injury. Second, we used a relatively small dose of vasopressin, less than is generally used in humans. Also, the effects of vasopressin infusion were observed only for a short-term period. Thus, the true clinical applicability is perhaps difficult to precise. Finally, we used capillary leakage as a marker for endothelium dysfunction in the experimental model; however, capillary leakage is not a “synonym” of organ dysfunction or damage. Indeed, histopathological and immunohistochemical analyses should be performed to evaluate the effects of vasopressin on organ damage or dysfunction.

## Conclusions

Vasopressin was proposed as a vasoactive drug for provisional hemodynamic optimization in the early phase of HS resuscitation. In this study, vasopressin restored the MAP while causing a significant decrease in the mesenteric blood flow. In the vasopressin group, increased capillary leakage was observed in the lung, which was markedly higher than that in the group resuscitated with RL plus blood; this increase was also associated with a significant decrease in pO_2_. Conversely, a decrease in capillary leakage was observed with vasopressin in the ileum. Lastly, we found that vasopressin use was associated with significantly higher base deficit and lactate levels when compared to the other groups.

In conclusion, vasopressin may be used as a bridge to definitive treatment for HS, but the overall findings of this experimental study focus on the possible critical side effects of vasopressin on metabolic parameters and endothelium permeability. Based on the available information and due to the small number of human studies on this issue, it is still controversial whether the use of vasopressin may positively impact on outcomes in uncontrolled bleeding patients. Thus, randomized controlled trials are needed to clarify the effects of vasopressin in HS [39, 47].

## Abbreviations

b.w.: Body weight
EB: Evans blue
HS: Hemorrhagic shock
MAP: Mean arterial pressure
MBF: Mesenteric artery blood flow
MVR: Mesenteric vascular resistance
pO_2_: Partial pressure of oxygen
RL: Ringer’s lactate
RLB: Ringer’s lactate and blood
S: Sham-operated
